# Identification of a novel necroptosis-related classifier to predict prognosis and guide immunotherapy in breast invasive carcinoma

**DOI:** 10.3389/fonc.2022.852365

**Published:** 2022-09-05

**Authors:** Qin Zhou, Yan Xu, Liang Shen, Xiaochen Yang, Li Wang

**Affiliations:** ^1^ Department of Breast Surgery, Affiliated Kunshan Hospital of Jiangsu University, Kunshan, China; ^2^ Department of Oncology, Kunshan Traditional Chinese Medicine Hospital, Kunshan, China

**Keywords:** breast invasive carcinoma, necroptosis, immunotherapy, tumor microenvironment, risk model

## Abstract

**Background:**

Necroptosis plays a crucial function in the progression of breast invasive carcinoma (BRCA). It may be triggered in cancer therapy to enhance anti-tumor immunity. However, the functions of necroptosis in tumors and its relationship with the tumor microenvironment (TME) remain largely unclear.

**Methods:**

Necroptosis-related genes (NRGs) were collated from high-quality literature reviews. A robust risk model was constructed to systematically evaluate the clinical value, functional status, effects exerted by the risk model on the TME, and the genomic variations based on the Gene Expression Omnibus (GEO) and The Cancer Genome Atlas (TCGA) meta-cohorts.

**Results:**

A risk model was constructed which comprised of six NRGs, including TNF receptor-associated factor 5 (TRAF5), Toll-like receptor 3 (TLR3), a riboflavin kinase (RFK), Fas ligand (FASLG), Fas-associated protein with death domain (FADD), and baculoviral IAP repeat-containing 3 (BIRC3). The stability and accuracy of the risk model were demonstrated for both the training and validation cohorts and its utility as an independent prognostic model for BRCA was verified. Patients in the low-risk group exhibited “hot” tumors having active immune and cell killing functions, while those in the high-risk group showed “cold” tumors having active tumor proliferation and immunosuppression. Moreover, patients in the high-risk group had a greater number of CNV events in their genome, while the somatic mutations were fewer. Furthermore, patients in the low-risk group showed high sensitivity toward immunotherapy and chemotherapy.

**Conclusion:**

A reliable risk model based on NRGs to assess patient prognoses and guide clinical decision-making was constructed and validated. Our findings may contribute to the understanding of necroptosis and aid clinical management, along with precision treatment in BRCA.

## Introduction

Breast invasive carcinoma (BRCA) is a commonly prevalent cancer type and only the second top-ranking cause of tumor-related deaths in women worldwide (1). Statistics of the last five years on the prevalence of BRCA indicate that nearly 11% of the total reported BRCA cases worldwide were from China (2). Conventional therapies for BRCA include surgery, endocrine therapy, chemotherapy, targeted therapy, immunotherapy, and radiotherapy (3). Rapid advances in medicine have led to significant improvements in the prognosis of BRCA. However, patients with advanced BRCA often show resistance to treatment, and consequently, poor clinical outcomes (4). Accurate prognostic predictions for patients with BRCA can potentially improve their survival rates and facilitate the development of tailored treatment by physicians. Hence, new prognostic markers need to be identified. In recent years, some exciting developments have occurred in this field. Several reports reveal that tumor mutation burden (TMB) can predict patient prognoses in several cancer types, thereby making it a promising biomarker of sensitivity toward the immune checkpoint inhibitors (5, 6).

Necroptosis, a novel type of programmed cell death, was first reported in 2005 (7). It is a genetically programmed, lysogenic apoptosis mechanism, that is regulated in a caspase-independent manner. It is an alternative mode of apoptosis that overcomes resistance, along with triggering and enhancing anti-tumor immunity in cancer therapy (8, 9). In the onset of necroptosis, the activation of the protein kinases, including the Recombinant Receptor Interacting Serine Threonine Kinase 3 (RIPK3) and RIPK1, is implicated. This is followed by phosphorylation of the executioner molecule, mixed lineage kinase domain-like (MLKL), thereby inducing a rupture in the cell membrane (10–12). In cancer, necroptosis is a double-edged sword. If, on the one hand, apoptosis is not induced, necroptosis can provide an alternative to apoptosis, thereby eliciting a strong adaptive immune response and halting tumor progression. On the other hand, in the case wherein the induced inflammatory responses promote tumorigenesis and metastases, necroptosis can elicit the formation of an immunosuppressive tumor microenvironment (TME) under specified circumstances (8). Thus, to determine the role of necroptosis for patient prognoses, immune regulation, and therapy for different cancer types, a better understanding of the mechanisms underlying necroptosis and their physiological and pathological functions is warranted.

In the present study, 33 necroptosis-related genes (NRGs) were systematically assessed and their patterns in BRCA were analyzed using multi-omic data. Thus, seven independent prognosis-related NRGs were selected after Cox regression and then modeled using the robust iterative least absolute shrinkage and selection operator (LASSO) regression for BRCA. Next, we systematically assessed the prognostic model’s stability and accuracy in both the external validation and the training cohorts. The biological functions, TME, and genomic variations in the prognostic model were evaluated in detail. Finally, the value of the prognostic model was determined and its clinical applicability in chemotherapy and immunotherapy of BRCA was evaluated.

## Methods

### Data extraction from online databases

The clinical information of BRCA patients and their corresponding transcriptomic RNA sequences, Mutect2 mutation, HumanMethylation450 arrays, and copy number variation (CNV) data were extracted using the GDC application programming interface from The Cancer Genome Atlas (TCGA) (https://cancergenome.nih.gov/). Patients with incomplete clinical data or those who were lost to follow-up were excluded, following which, a total of 887 BRCA samples were included. We then standardized and normalized the primary data to reduce the heterogeneity between samples, the raw fragments per kilobase million (FPKM) sequence data were normalized to transcript per million (TPM) units and used as the training cohort. Additionally, three datasets from the GPL580 platform of the Gene Expression Omnibus (GEO) database, GSE20685, GSE20711, and GSE42568, were collated(https://www.ncbi.nlm.nih.gov/geo/). The microarray data were merged and normalized from the three GEO datasets and the batch effects were eliminated using the combat function of the “sva” package (13), resulting in the collection of the meta-data of 519 BRCA patients having entire clinical details in the validation cohort. In addition, we obtained the publicly available immunotherapy data with complete clinical and transcriptomic information, resulting in a cohort of 298 patients with advanced uroepithelial cancer (Imvigor210) who underwent anti-PD-L1 immunotherapy (14).

### Generation and validation of the NRG-related risk model

The model was trained based on the TCGA cohort. First, for BRCA, all the independent prognostic factors were screened using univariate COX regression, and only the significant (P< 0.05) genes were included for further analysis. The least absolute shrinkage and selection operator (LASSO) penalized Cox proportional risk model was employed to identify the best prognostic model. To avoid overfitting, a five-fold cross-validation was performed. Considering random sampling for cross-validation, a total of 250 iterations were performed to identify the most stable prognostic model and the most frequently occurring model among the 250 iterations was the final prognostic model. The RiskScore was computed as follows:


$$Riskscore=\sum iCoefficient(mRNA_{i})\times Expression(mRNA_{i})$$

We also constructed the RiskScore in both GEO and Imvigor210 cohort based on the same formula. The consistency index (c-index) was computed using “survcomp” to assess the predictive power of the RiskScore in both the training and validation cohorts; the larger the c-index, the more accurate the model (15). The median RiskScore was used to divide patients into the high- and low-risk groups. Moreover, the prognostic value of the risk model was systematically assessed using the following analyses: Kaplan-Meier survival curves, time-dependent receiver operating characteristic (ROC) curves, and multivariate and univariate Cox regression.

### Functional enrichment and immune infiltration analyses

We explored the potential biological function of NRGs in the TCGA cohort. The pathway activities associated with the samples were assessed by single-sample gene set enrichment analysis (ssGSEA) using “gsva” in R. Gene markers for angiogenesis, epithelial-mesenchymal transition (EMT), myeloid inflammation, as well as other molecular markers of immune-related pathways, were collated from previously published high-quality literature (16–19). Molecular markers for hypoxia were obtained from the Msigdb database (www.plob.org/tag/msigdb) (20). Details on the gene markers are listed in **Table S1**. In addition, for making a comparison between the risk groups and screening the significant (P< 0.05) Kyoto Encyclopedia of Genes and Genomes (KEGG) pathways, a gene set enrichment analysis (GSEA) was performed using the GSEA software (version 4.2.2).

The abundant infiltration of 22 different immune cells in the tumor samples was determined using the R package, “CIBERSORT” (cibersort.stanford.edu) (21). In addition, the immune activity and tumor purity of the samples were assessed using the Estimate algorithm (22).

### Landscape of genomic variations between the groups

The genomic variations between the high and low-risk group was evaluated in the TCGA cohort. To compare the differences in the tumor mutation burden between the two risk groups, we processed the mutation data using the “maftools” package in R and calculated the total number of mutations in the samples. Genes with a minimum number of mutations > 30 were subsequently selected, and the differences in the mutation frequencies between the risk groups were compared using the chi-square tests and visualized using maftools (23). CNV data were processed using Gistic (version: 2.0) of the Genepattern webtool (www.genepattern.org) and significant amplifications and deletions were identified; the CNV landscape was visualized using the R package, Circos (circos.ca).

### Assessment of the clinical significance of the risk model

First, the median inhibitory concentration (IC50) for four first-line BRCA drugs (gemcitabine, docetaxel, paclitaxel, and doxorubicin) was calculated in the validation and training cohorts using ridge regression algorithm of the pRRophetic package; the smaller was the IC50 value, the greater was the sensitivity to the drugs (24). Differentially expressed genes (DEGs) between the risk groups were the putative therapeutic targets, we identified the DEGs based on the threshold fold change>2 and FDR<0.05 using the “limma” R package. CMap database was used to determine their putative target compounds (https://clue.io/). After querying the top 150 upregulated and downregulated DEGs, we predicted the putative small molecule compounds (25). Moreover, the patient responses to immunotherapy were predicted using the Tumor Immune Dysfunction and Exclusion (TIDE) online tool (http://tide.dfci.harvard.edu) (26). The unsupervised subclass mapping algorithm assessed patient responses to anti-PD1 and anti-CTLA-4 immunotherapies(https://cloud.genepattern.org/gp/). Finally, the predictive utility of the RiskScore was verified in the Imvigor210 immunotherapy cohort.

### Specimen collection

67 cases of surgically resected cancerous tissues of breast cancer patients diagnosed and treated in our hospital from September 2015 to April 2016, with a median age of 42 ~ 73 years and a mean age of (56.19 ± 9.1) years were selected. **Inclusion criteria:** (1) All were diagnosed with breast cancer by postoperative pathological examination; (2) All did not receive radiotherapy or chemotherapy before surgery; (3) Clinical data were complete. **Exclusion criteria: (1)** Combination of chronic systemic diseases; (2) Combination of other malignant tumors. Our ethics committee approved the study (IEC-C-001-A04-V3.0), supervised by our ethics committee, and the subjects all signed an informed consent form while enjoying the right to information.

### Immunohistochemistry staining

After regular dewaxing and rehydration of the tissue in paraffin sections, the sections were closed with 3% H2O2 for 10 min to prevent non-specific staining; primary antibody was added dropwise and incubated overnight at 4°C. The next day, the overnight sections were rewarmed at 4°C, washed 3 times with double distilled water, soaked and moistened with washing buffer, added dropwise with secondary antibody working solution, and stained at room temperature for 1h. The IHC staining score was calculated by multiplying the staining intensity (0, negative; 1, mild; 2, moderate; 3, strong) and the proportion of positive cells (0, negative; 1,<10%; 2, ≥10% and<33%; 3, ≥33% and<66%; 4, ≥66%) for each tissue in turn according to the staining on the tissue sections. The expression of HDAC11 in breast cancer tissues was classified as low (0-6) or high (8-12) expression according to the tissue microarray immunohistochemical staining score.

### Bioinformatics and statistical analyses

All statistical analyses and graph plotting were done on R (version: 4.04). Comparisons between groups were performed using the Wilcoxon test. The Kaplan-Meier plotter was used to generate survival curves and statistically significant differences were assessed using the log-rank test. Time-dependent receiver operating characteristic curves (tROC) were plotted using ‘survivalROC’. The R package, ‘survival’ was used to perform multivariate and univariate Cox regression analyses; the ‘rms’ package was used to construct the nomogram and plot the calibration curves; decision curve analysis (DCA) was performed using the DCA package (27). Two-tailed p< 0.05 was the statistical significance threshold unless stated otherwise.

## Results

### The landscape of genomic variations in NRGs in BRCA

**Figure 1:**
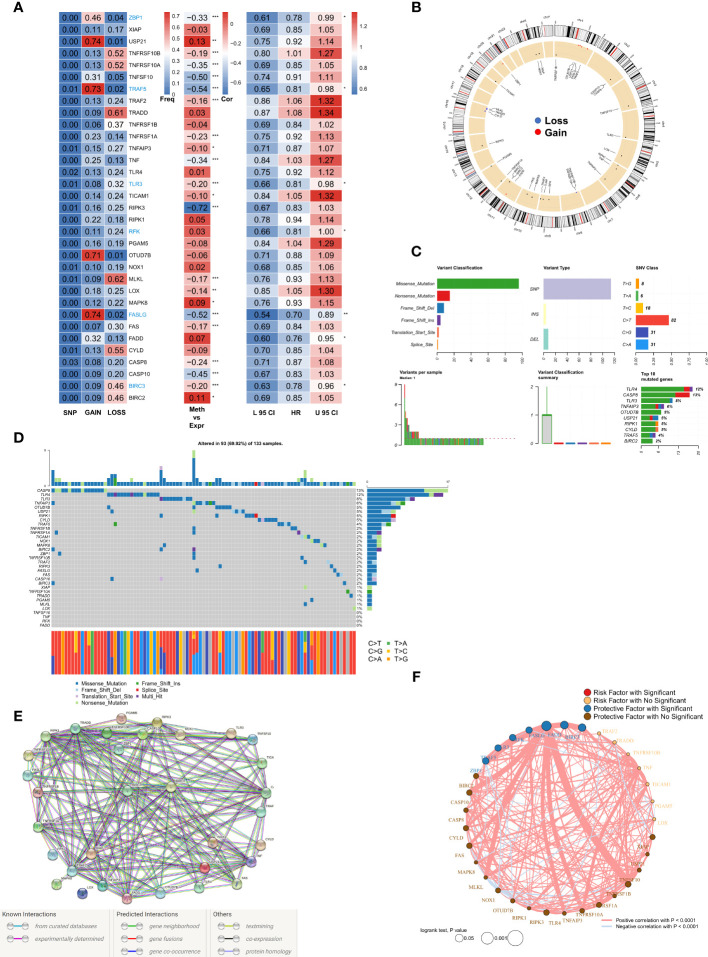
Genomic mapping for NRGs in BRCA **(A)**. Heat map showing genomic changes and hazard ratios of NRGs in TCGA-BRCA cohort; from left to right: correlation between mutation and CNV frequencies of NRGs, modifications in DNA methylation and expression of NRGs, and a univariate Cox regression analysis showing risk ratios for NRGs; *p < 0.05, **p < 0.01, ***p < 0.001; **(B)**. Circle plot demonstrating CNV events in NRGs on the chromosomes; **(C)**. Summary of CNV events in NRGs in TCGA-BRCA cohort; **(D)**. Oncoplot showing the mutational map of NRGs; **(E)**. PPI network of NRGs based on STRING; **(F)**. Correlation network of NRGs.

A total of 33 NRGs from previously published high-quality reviews were collated and details of the selected genes are listed in **TableS2**. Specifically, first, the multi-omics profiles of NRGs in TCGA-BRCA patients were summarized (**Figure 1A**). The frequency of single nucleotide variations (SNVs) in NRGs was low but that of CNVs, encompassed a wide range, especially for USP21, TRAF5, OTUD7B, and FASLG, which hinted that CNVs may exert dominant effects in NRG regulation as compared to gene mutations. In addition, a significant correlation was observed between methylation regulators and gene expressions of NRGs, especially for RIPK3, CASP10, TNFSF10, TRAF5, and FASLG. Seven genes (ZBP1, TRAF5, TLR3, RFK, FASLG, FADD, and BIRC3) were found to exert significant protective roles, and subsequently, these factors were used for the construction of risk models. The results of the Cox regression analysis are listed in **Table S3**. **Figure 1B** shows the CNV profiles of NRGs on chromosomes. Next, the mutation profiles of NRGs (**Figure 1C**) were summarized, and CASP8 and TLR4 were found to be the two most frequently mutated genes. Moreover, the most common mutation was missense; SNV was the most common mutation type with cytosine to thymine change, being the most frequent. The waterfall diagram shows the mutation profiles of NRGs in patients (**Figure 1D**). We then queried the NRGs based on the confidence level of 0.9 using the STRING database (string-db.org) and obtained a protein interaction network (**Figure 1E**); BIRC2 and BIRC3 genes were identified as the hub. Finally, we mapped the correlation network of NRGs and selected the significantly (P< 0.0001) positively correlated pairs (**Figure 1F**).

### Construction of the NRG-related risk model

**Figure 2:**
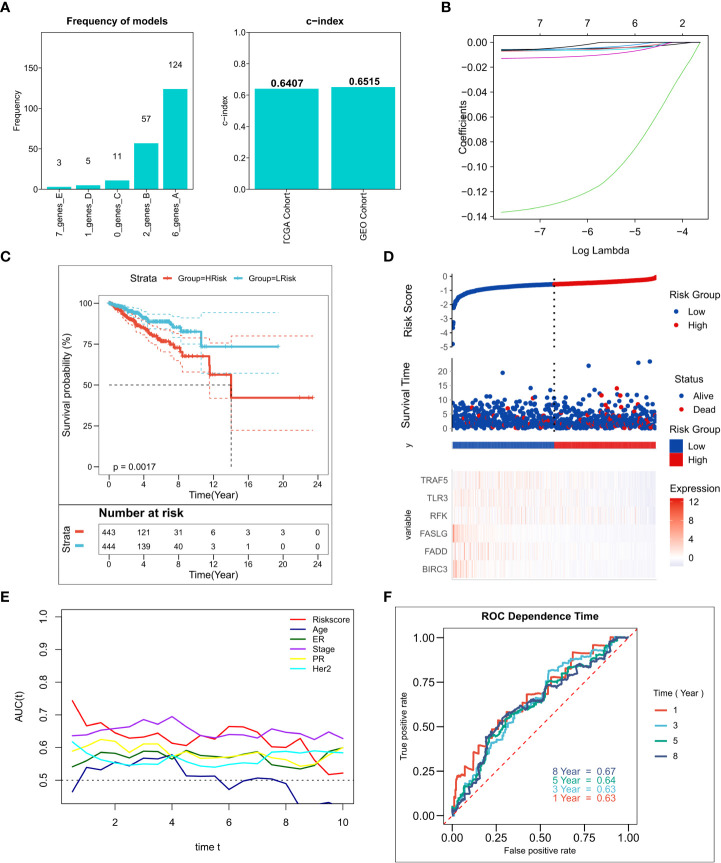
Construction of the NRG-related risk model **(A)**. Screening the best LASSO model; left: frequency of different gene combinations in the LASSO Cox regression model, right: c-index of the best model in TCGA and GEO cohorts; **(B)**. LASSO Cox regression model to identify the most robust nine-gene signature marker having an optimal λ value of 0.00547; **(C)**. KM survival curves for the high- and low-risk groups in the TCGA cohort. **(D)**. Survival status of patients and expression of marker genes in TCGA cohort; **(E)**. tROC curves for risk models and clinical characteristics in TCGA cohort; **(F)**. 1-, 3-, 5-, and 8-year ROC curves for the RiskScore in TCGA cohort.

We performed 250 iterations of LASSO regression for screening the most important prognostic factors and constructing a stable risk model. The model containing six genes, including TRAF5, TLR3, RFK, FASLG, FADD, and BIRC3, was determined to be the most stable. It exhibited good accuracy in both the training and validation cohorts (TCGA: 0.6407; GEO: 0.6515) (**Figure 2A**). The model was constructed based on an optimal λ value of 0.00547 (**Figure 2B)**, and the RiskScore was computed as follows:


$$Risk\;Score=\sum^{}iCoefficient\left(mRNA_{i}\right)\times Expression\left(mRNA_{i}\right)$$

The LASSO coefficients for the model genes are provided in **Table S4**. Patients were classified into low-risk and high-risk groups based on the median RiskScore. The survival analysis suggested that patients in the high-risk group showed markedly lower survival relative to those in the low-risk group (**Figure 2C**; P = 0.0017). **Figure 2D–F** shows the distribution of RiskScore and the transcriptomic map of genes in the model for the TCGA cohort. Additionally, the tROC analysis showed that RiskScore and TNM staging were the best prognostic predictors (**Figure 1E**). Specifically, the model had 1-, 3-, 5-, and 8-year AUC values of 0.63, 0.63, 0.64, and 0.67, respectively (**Figure 1F**). The predictive utility of the model was assessed in the validation cohort. The survival analysis suggested significantly worse survival rates in the high-risk group (**Figure S1A**, P< 0.0001). **Figure S1B** shows the distribution of RiskScore and model genes in the GEO cohort. The 1-, 3-, 5-, and 8-year AUC values for the model in the validation cohort were 0.62, 0.67, 0.69, and 0.70, respectively (**Figure S1C**).

### Predictive independence of the risk model

**Figure 3:**
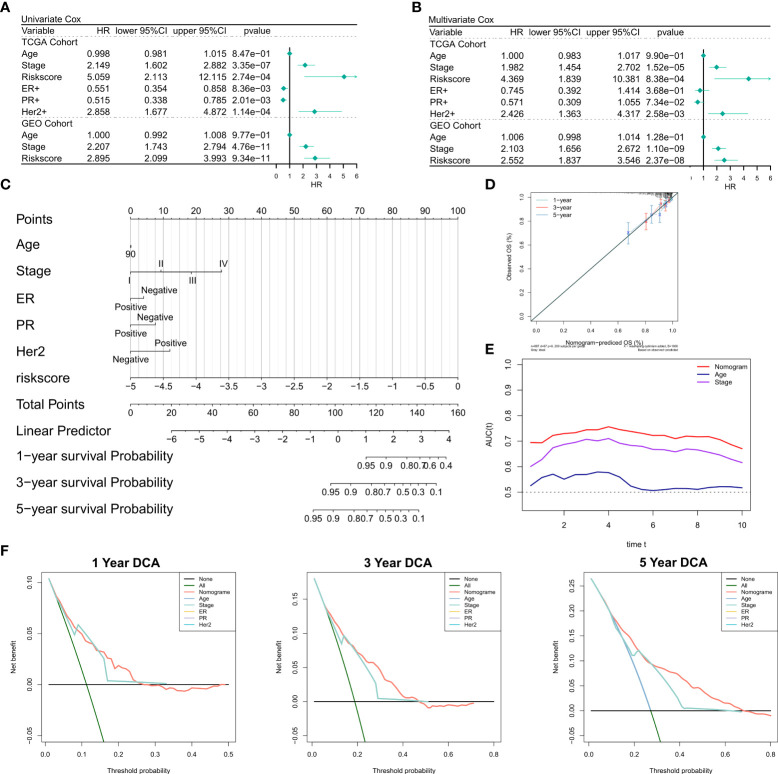
Validation of the NRG-related risk model **(A)**. Univariate Cox regression analysis for OS in TCGA and GEO cohorts; **(B)**. Multivariate Cox regression analysis for OS in TCGA and GEO cohorts; **(C)**. Nomogram for the NRG-related risk model; **(D)**. Calibration curves for the nomogram; **(E)**. tROC curves for the nomogram and clinical characteristics; **(F)**. 1-, 3-, and 5-year DCA curves for the nomogram.

First, we investigated the relationship of RiskScore and corresponding clinical characteristics [including AJCC TNM stage, estrogen receptor (ER), progesterone receptor (PR) and human epidermal growth factor receptor 2 (Her2)] with the prognoses of patients using multivariate and univariate Cox regression. The univariate Cox regression analysis suggested that RiskScore (hazard ratio [HR] = 5.509, P< 0.001), TNM stage (HR = 2.149, P< 0.001), ER+(HR = 0.551, P = 0.008), PR+ status (HR = 0.515, P = 0.002), and Her2+ status (HR = 2.858, P=0.0353) in the training cohort were significantly associated with patient prognoses (**Figure 3A**); RiskScore (HR = 2.858, P< 0.001) and TNM stage (HR = 2.207, P< 0.001) were significantly associated with patient prognoses (**Figure 3A**). After correction for other clinical characteristics, the results of the multivariate Cox regression confirmed that RiskScore remained an independent prognostic factor for the OS of patients (TCGA: HR = 4.389, P< 0.001; GEO: HR = 2.552, P< 0.001) (**Figure 3B**). Thus, RiskScore could serve as a prognostic marker for OS in BRCA patients. Given that the TCGA cohort consisted of more detailed molecular types of BRCA, we constructed the nomogram to better quantify the risk assessment of these patients (**Figure 3C**). The correction curves showed good 1-, 3-, and 5-year stability, as also the accuracy of the nomogram (**Figure 3D**). Moreover, the tROC analysis showed that the nomogram was a better predictor relative to clinical characteristics (**Figure 3E**). DCA was performed to assess the decision benefit of the nomogram and the results showed its good applicability in 1-, 3-, and 5-year risk assessment of patients with BRCA (**Figure 3F**).

### Functional enrichment analysis of the risk model

**Figure 4:**
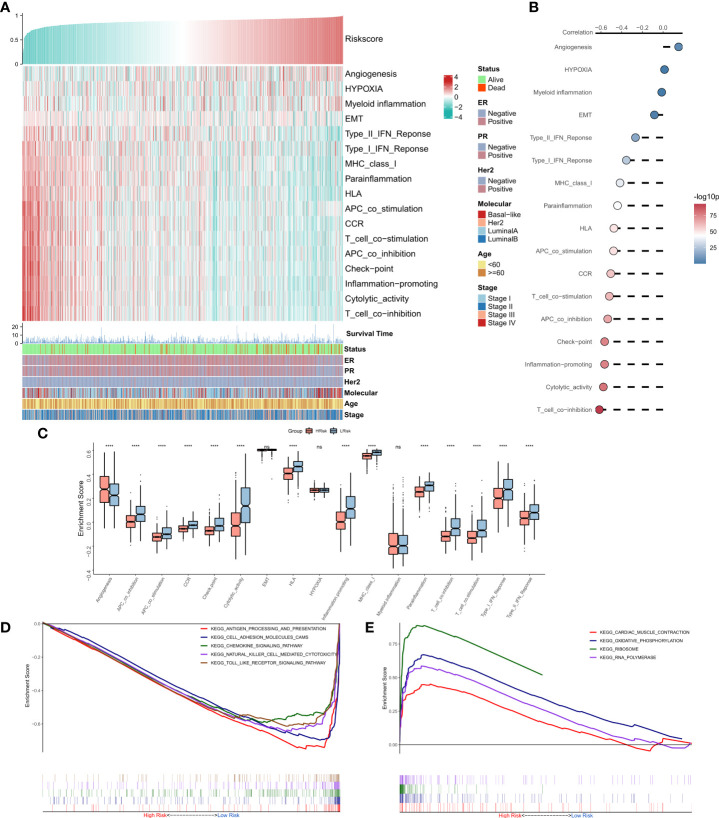
Functional analysis for the NRG-related risk model **(A)**. Heat map showing the correlation between RiskScore, activities of biological pathways, and clinical characteristics; **(B)**. Correlation analysis between RiskScore and biological pathways; **(C)**. Box plots showing the differences in the activities of the biological pathways between the high-risk and low-risk groups; **(D)**. GSEA plot showing the top five pathways of interest in the high-risk group; **(E)**. GSEA plot showing the top four pathways of interest in the low-risk group ****p < 0.0001. ns, p > 0.05.

The correlations between RiskScore and some typical biological pathways were assessed. The heat map shows the relationship between RiskScore, activities of the biological pathways, and clinical characteristics (**Figure 4A**). The results of the correlation analysis between RiskScore and biological pathways are shown on the right of the heat map (**Figure 4B**). We found that angiogenesis was significantly positively correlated with RiskScore, while all immune-related pathways except myeloid inflammation were negatively correlated with RiskScore. Consistently, we observed that angiogenesis was significantly higher in the high-risk group, while the immune-related pathways were markedly enhanced in the low-risk group (**Figure 4C**). Further, GSEA showed that the pathways related to antigen presentation, chemokine secretion, and Toll-like receptor signaling (**Figure 4D**) were markedly upregulated in the low-risk group, while the high-risk group was significantly enriched in pathways associated with the ribosome and RNA splicing (**Figure 4E**). In summary, these results suggested that tumor angiogenesis and DNA replication were hyperactive in the high-risk group, while cell-killing and immune activities were markedly enhanced in the low-risk group.

### The immune landscape of the risk model

**Figure 5:**
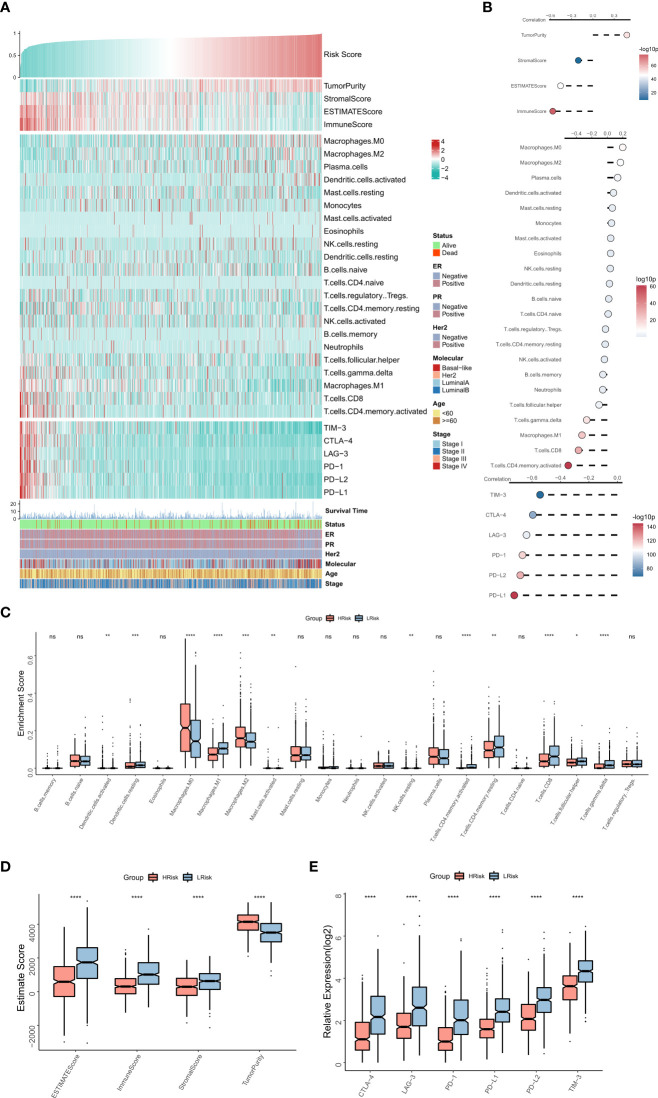
The immune landscape of the NRG-related risk model **(A)**. Heat map showing the correlations between RiskScore, EstimateScore, the abundance of immune cell infiltration, immune checkpoint expression, and clinical characteristics; **(B)**. from top to bottom: correlation between RiskScore and EstimateScore, between RiskScore and immune cell infiltration abundance, and between RiskScore and immune checkpoint expression; **(C)**. Box plot showing the differences in the abundances of immune cell infiltration between the high-risk and low-risk groups; **(D)**. Box plot showing the differences in EsimateScore between the high-risk and low-risk groups; **(E)**. Box plot showing the differences in immune checkpoint expression between the high-risk and low-risk groups *p < 0.05; **p < 0.01; ***p < 0.001 **** p < 0.0001. ns, p > 0.05.

The association between the immune landscape and RiskScore was assessed in detail. The heat map shows the relationships between RiskScore, EstimateScore, the infiltration abundance of immune cell types, typical immune checkpoints (including PD-L1, PD-L2, PD-1, CTLA-4, LAG-3, and TIM-3), and clinical characteristics (**Figure 5A**). The corresponding correlations are shown on the right of the heat map (**Figure 5B**). The box plot showed an enhanced abundance of M0 macrophages, M2 macrophages, and activated dendritic cells in the high-risk group, while M1 macrophages, CD8T cells, and Gamma delta T cells are markedly upregulated in the low-risk group (**Figure 5C**), consistent correlational findings. Further, a significant positive correlation was observed between RiskScore and tumor purity, while those between EstimateScore, ImmuneScore, and expressions of the six immune checkpoints showed a substantial negative association. Consistently, box plots showed that tumor purity was elevated in the high-risk group, whereas EstimateScore, ImmuneScore, and expressions of the six immune checkpoints were elevated in the low-risk group (**Figures 5 D, E**). Overall, these results suggested that the high-risk group showed immunosuppression of antitumor effects, while the low-risk group exhibited active anti-tumor immune activity and cell-killing functions.

### Genomic mutations of subtypes

**Figure 6:**
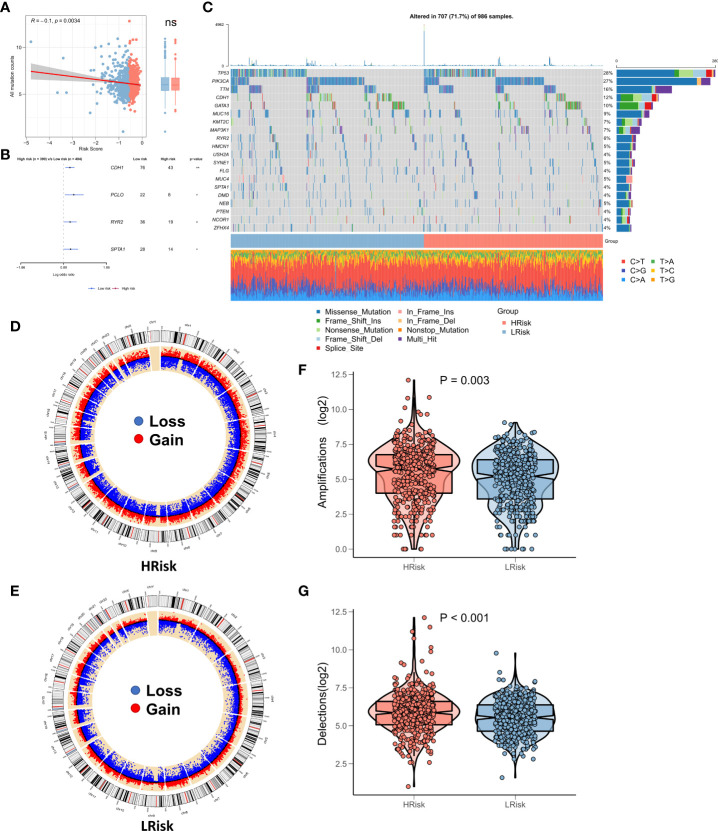
Landscape of genomic variations for the NRG-related risk model **(A)**. Correlation between RiskScore and TMB; **(B)**. Forest plot showing genes with significant differences in mutations between the high-risk and low-risk groups; **(C)**. Oncoplot showing the significantly mutated genes between the high-risk and low-risk groups; **(D)**. Circle plot showing the CNV landscape in the high-risk group; **(E)**. Circle plot showing the CNV landscape in the low-risk group; **(F)**. Box plot showing the differences in the number of chromosomal deletions between the high-risk and low-risk groups; **(G)**. Box plots showing the differences in the number of chromosome amplifications between the high-risk and low-risk groups *p < 0.05; **p < 0.01. ns, p > 0.05.

Recent studies show a strong correlation of TMB with anti-tumor immunity and immunotherapeutic efficacy, as high TMB generates a greater proportion of mutated peptide fragments that can be recognized by the immune system, resulting in an enhanced anti-tumor immunity function. Considering the clinical significance of TMB, we examined the correlation between TMB and RiskScore and the results showed a significant negative correlation between the two (correlation = -0.10, p = 0.034). There was no significant elevation in TMB in the low-risk group (**Figure 6A**). The forest plot shows that CDH1, PCLO, RYR2, and SPTA1 were significantly more frequently mutated in the high-risk group, whereas PIK3CA, FAT3, FAT4, and LRP1B were commonly mutated in the low-risk group (**Figure 6B**). In addition, the oncoplot showed the detailed mutational landscape in the high- and low-risk groups in BRCA (**Figure 6C**). As CNVs too can cause chromosomal variations, we further evaluated the correlation between RiskScore and CNVs. We found a greater number of CNV events (deletion and amplification) in the high-risk group (**Figure 6D**) relative to the low-risk group (**Figure 6E**). Box plots showed a significant increase in both deletion and amplification events in the high-risk group (**Figures 6F, G**).

### The role of the risk model in guiding clinical decision-making

**Figure 7:**
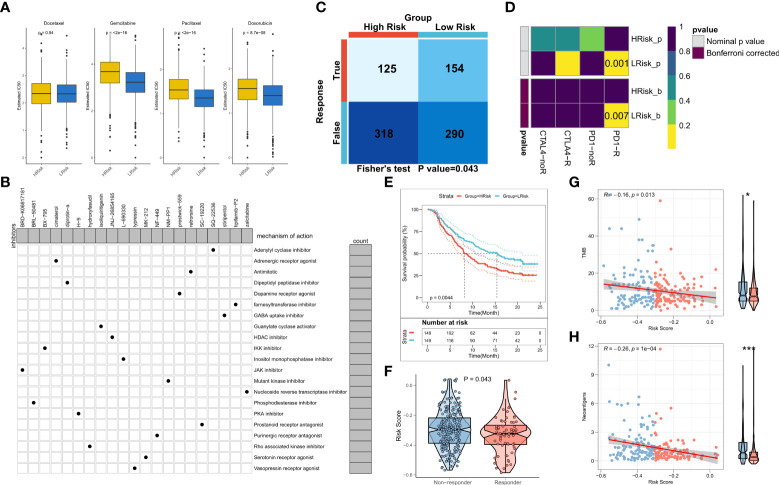
NRG-related risk model in guiding decision-making for clinical treatment **(A)**. Box plot showing predicted IC50 values of four commonly used drugs between the two risk groups; **(B)**. Oncoplot showing the target small molecule compounds, wherein the horizontal axis represents the name of the small molecule inhibitor, while the vertical axis represents the specific biological pathway targeted by the small molecule inhibitor; **(C)**. TIDE algorithm for predicting immunotherapeutic responses in the high-risk and low-risk groups; **(D)**. Subclass mapping for predicting sensitivity to anti-PD1 and anti-CTLA4 treatment in patients belonging to the high-risk and low-risk groups; **(E)**. KM survival curves for the high-risk and low-risk groups in the IMvigor 210 cohort; **(F)**. Box plot showing the differences in RiskScore between patients in the treatment-responsive and non-responsive groups in IMvigor 210; **(G)**. Correlation between RiskScore and TMB in the IMvigor210 cohort; **(H)**. Correlation between RiskScore and neoantigens in the IMvigor 210 cohort *p < 0.05; ***p < 0.001.

We first assessed differences in the sensitivity of BRCA patients to different chemotherapeutic agents and the findings suggested that the patients in the low-risk group of the TCGA cohort had enhanced sensitivity to gemcitabine, paclitaxel, and doxorubicin (**Figure 7A**). Same results in the validation cohort were obtained (**Figure S2A**). Overall, patients in the low-risk group exhibited a greater sensitivity towards chemotherapy and the DEGs between the risk groups could be potential targets of small molecule compounds. Thus, DEGs were queried on the Clue database for the identification of putative small molecule drugs. The waterfall diagram shows the potential 21 small molecule drugs and their corresponding target biological pathways (**Figure 7B**). Our results for immune landscape and genomic variations between groups suggested that RiskScore may be significantly related to the efficacy of immunotherapy. Hence, we assessed the patient response rates to immunotherapy using the TIDE algorithm (tide.nki.nl). Patients in the low-risk group of the TCGA cohort were more likely to respond to immunotherapy (P = 0.043, **Figure 7C**). Although the number of responses to immunotherapy was higher in the low-risk group of the training cohort, it was not statistically significant (P = 0.073, **Figure S2B**) Subsequently, subclass mapping results suggested that patients in the low-risk group showed enhanced sensitivity to anti-PD1 therapy in both TCGA and GEO cohorts (TCGA: false discovery rate [FDR] = 0.007, GEO: FDR = 0.001) (**Figure 7D**; **Figure S2C**). Finally, we computed the RiskScore in a well-established immunotherapy cohort, which showed significantly worsened survival in patients belonging to the high-risk group (P = 0.044, **Figure 7E**). The RiskScore was significantly higher in patients who did not respond to immunotherapy (**Figure 7F**). We then evaluated the relationship between TMB and neoantigens and RiskScore in the immunotherapy cohort, which showed that RiskScore was negatively correlated with TMB and neoantigen counts, both of which were markedly elevated in the low-risk group (**Figures 7G, H**). Thus, these results confirmed that the generated risk model was a powerful tool for guiding immunotherapy in patients with BRCA.

### Validation of key NRGs in the clinical samples

**Figure 8:**
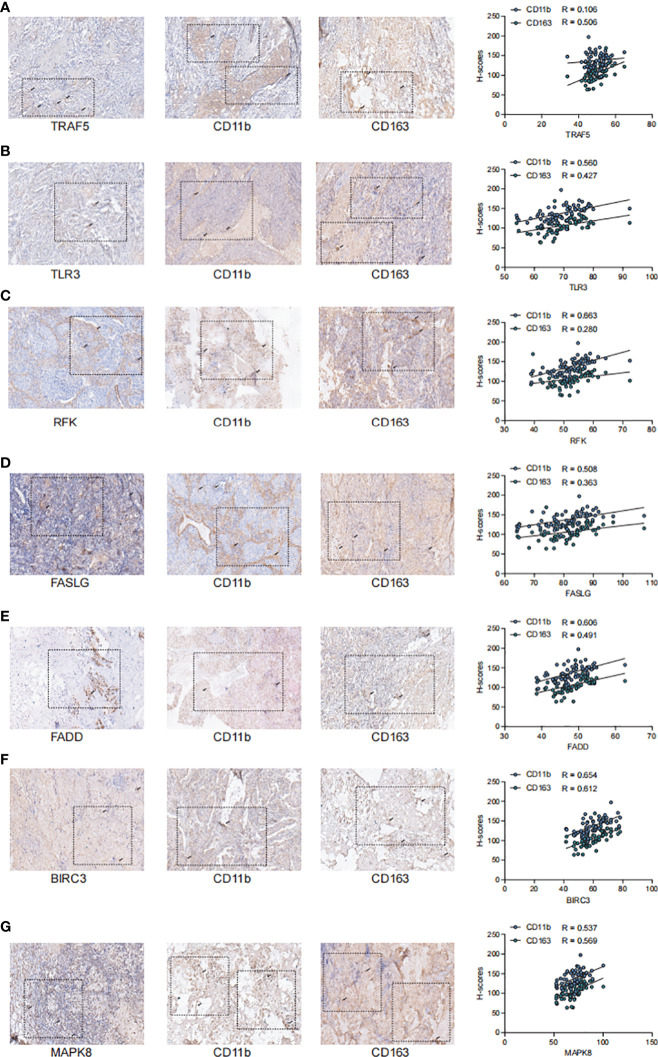
TRAF5, TLR3, RFK, FASLG, FADD, BIRC3, MAPK8 showed significant positive correlation with the staining intensity of TME markers **(A)**. Staining intensity of TRAF5 with TME-associated Markers CD11b and CD163, correlation coefficient of TRAF5 with CD11b is 0.106 and with CD163 is 0.506; **(B)**. Staining intensity of TLR3 with TME-associated Markers CD11b and CD163, correlation coefficient of TLR3 with CD11b is 0.560 and the correlation coefficient with CD163 was 0. 427; **(C)**. Staining intensity of RFK with TME-associated Markers CD11b and CD163, the correlation coefficient of RFK with CD11b was 0.663 and the correlation coefficient with CD163 was 0.280; **(D)**. FASLG with TME-associated Markers CD11b and CD163 correlation coefficient, the correlation coefficient of FASLG with CD11b was 0. 508 and with CD163 was 0.363; **(E)**. Correlation coefficient of FADD with TME-related Marker CD11b and CD163, the correlation coefficient of FADD with CD11b was 0.606 and with CD163 was 0.491; **(F)**. Correlation coefficient of BIRC3 with staining intensity of TME-associated Markers CD11b and CD163, the correlation coefficient of BIRC3 with CD11b was 0. 6540 and with CD163 was 0.612; **(G)**.The correlation coefficient of MAPK8 with TME-associated Markers CD11b and CD163, the correlation coefficient of MAPK8 with CD11b was 0.537 and with CD163 with a correlation coefficient of 0.569.

To further validate the stability of the model, we collected tumor tissues from 67 breast cancer patients from the clinic and examined the correlation between the staining intensity of the screened Markers: TRAF5, TLR3, RFK, FASLG, FADD, BIRC3, MAPK8 and the TME-related Markers CD11b and CD163 by immunohistochemical staining (**Figure 8**). We observed that the staining intensity of TRAF5, TLR3, RFK, FASLG, FADD, BIRC3. The staining intensity of MAPK8 showed a significant positive correlation with the staining intensity of CD11b and CD163 in TME (P< 0.01),the correlation coefficient of TRAF5 with CD11b was 0.106 and with CD163 was 0.506; the correlation coefficient of TLR3 with CD11b was 0.560 and with CD163 coefficient was 0.427; RFK had a correlation coefficient of 0.663 with CD11b and 0.280 with CD163; FASLG had a correlation coefficient of 0.508 with CD11b and 0.363 with CD163; FADD had a correlation coefficient of 0.606 with CD11b and 0.491 with CD163 0.491; BIRC3 had a correlation coefficient of 0.6540 with CD11b and 0.612 with CD163; MAPK8 had a correlation coefficient of 0.537 with CD11b and 0.569 with CD163. Overall, 7NRGS was positively correlated with TME, which is consistent with our results.

## Discussion

In the present study, we constructed a prognostic model for BRCA patients using a robust LASSO algorithm based on NRGs. In addition, the associations of the model with the biological functions, immune microenvironment, and genomic variations in cancer progression were systematically assessed and the value of the prognostic model in guiding clinical treatment decisions was examined. We confirmed the suitability and accuracy of the constructed prognostic model for predicting survival in BRCA patients in the external validation and the training cohorts. Tumor angiogenesis and DNA replication were active, whereas immune and cell-killing activities were hyperactive in the low-risk group. Moreover, immune microenvironment analysis demonstrated that immune function and anti-tumor immunity were more active in BRCA patients having low RiskScores. Genomic variation analysis suggested a significantly higher frequency of mutations of CDH1, PCLO, RYR2, and SPTA1 in the low-risk group. Moreover, chromosomal amplification and deletion events were also significantly higher in the high-risk group. For clinical applicability, we determined that patients with BRCA in the low-risk group were more sensitive to chemotherapeutic agents. Finally, we predicted better immunotherapeutic responses among BRCA patients having low RiskScores by TIDE and subclass mapping algorithms, along with determining the predictive performance of the risk model in an external immunotherapy cohort.

Apoptosis is strongly associated with cancer progression, metastasis, and treatment response. Inhibiting apoptosis enhances tumor metastasis and resistance of malignant cells to chemotherapy (28, 29). Ferroptosis, pyroptosis, and necroptosis are emerging forms of apoptosis. As most tumors are innately resistant to apoptosis, the induction of apoptosis mechanisms is emerging as a new strategy for cancer treatment (9). Existing evidence confirms the predictive value of pyroptosis and ferroptosis for predicting the prognosis of BRCA (30, 31). In this study, for the first time, we focus on necroptosis as an alternate apoptosis mechanism. The NRGs-based risk model showed excellent predictive performance in both the training and external validation cohorts, with a significant reduction in survival rates among the high-risk patients, which suggested that NRGs may exert important effects in precision medicine for BRCA.

To examine the functional biological mechanism underlying the survival differences, the correlation between the risk model and biological pathways was analyzed. We found that angiogenic activity was significantly higher in the high-risk group. Previous studies report that active angiogenesis is critical for tumor growth and metastasis. It is associated with the suppression of immune functions. Inhibition of angiogenesis is also a promising therapeutic target for impeding tumor growth (32–34). However, immune-related pathways, including those involved in cell killing, CCR, antigen presentation, interferon response, and myeloid immunity were found to be more active in the low-risk group, which suggested that antigen presentation, anti-tumor immunity, and cell killing are more potent in this risk group (35–37). In addition, GSEA suggested active ribosomal functions and RNA replication in patients belonging to the high-risk group, as also elevated levels of antigen presentation, chemokine Toll-like receptor signaling, and natural killer cell activity in patients in the low-risk group, which suggested active tumor proliferation in patients in former and immune hyperfunction in the latter (38–40). Overall, the aforementioned findings suggested that tumor proliferation and metastasis are stronger in patients in the high-risk group causing significantly poorer survival in these patients; those in the low-risk group showed stronger anti-tumor immunity, which may contribute to resistance to treatment.

As TME and immune activity are strongly associated with cancer treatment and patient prognoses (41, 42), we assessed differences in TME and immune activity between the low-risk and the high-risk groups. Notably, the low-risk group had higher immune scores and immune checkpoint activity, which suggested that these patients were more immunocompetent. Increased abundance of M2 macrophages, M0 macrophages, and activated dendritic cells in the high-risk group was consistent with the findings of our previous study (43). In contrast, the abundance of M1 macrophages, CD8T cells, and Gamma delta T cells in the low-risk group suggested that these patients exhibited stronger anti-tumor immunity (43, 44). These results indicated a greater probability of occurrence of immunosuppressed ‘cold’ tumors with a weaker anti-tumor response in the patients belonging to the high-risk group, ultimately leading to a poorer prognosis. In contrast, patients in the low-risk group developed immunocompromised ‘hot’ tumors, thereby showing better prognoses.

TMB is a biomarker of immunotherapeutic responses. In general, higher TMB results in the production of more neo-antigenic peptides, that are recognized by the immune system, thereby allowing for enhanced sensitivity to immunotherapy; however, there is heterogeneity in its predictive efficacy for different tumors (6, 45). Our findings suggested that RiskScore was significantly negatively associated with TMB. The TMB was not substantially elevated in the low-risk group, which suggested that RiskScore could robustly identify patients with immunologically active ‘hot’ tumors relative to TMB. We also analyzed the CNV events in patients in the TCGA-BRCA cohort. Patients in the high-risk group had a greater proportion of chromosomal amplification and deletion events. Previous studies show that somatic structural rearrangement events in chromosomes actively drive oncogenesis, thereby leading to greater tumor heterogeneity and chemoresistance (46–48). These results suggested that patients in the high-risk group may not respond well to treatment and that those in the low-risk group may be more sensitive to immunotherapy and chemotherapy.

As our previous findings strongly suggested that patients in the low-risk group exhibited enhanced sensitivity to treatment, we analyzed the sensitivity of the patients in both BRCA risk groups towards chemotherapy and immunotherapy. Both in the validation and training sets, it was confirmed that the patients in the low-risk group exhibited elevated sensitivity to gemcitabine, paclitaxel, and doxorubicin. In addition, TIDE and subclass mapping algorithms also predicted that patients in the low-risk group showed enhanced sensitivity to PD1 immunotherapy. Moreover, in an external immunotherapy cohort, the patients in the low-risk group were more sensitive to PD-L1 treatment and had a longer survival duration. This may be attributed to the elevated TMB and neoantigen counts among patients in the low-risk group. In conclusion, these results confirmed that the risk model used in this study was a powerful tool for guiding the treatment of patients with BRCA in clinical settings.

The necroptosis model can be simply implemented based on PCR-based assay, suggesting the potential for clinical translation and implementation of this study. Nevertheless, this study has some limitations. First, due to the paucity of data, only inter-patient heterogeneity was considered; intratumoral heterogeneity remained unaccounted for. Second, although we have used some algorithms for the assessment of the accuracy of this risk model in predicting the sensitivity of patients towards chemotherapy and immunotherapy, further validation in a prospective cohort study and clinical data are required. Third, some commonly used biochemical and test indicators are seriously inadequate in the public database, this may obscure potential associations between NRG models and certain variables and affect clinical implementation. Finally, although we preliminarily confirmed the negative correlation between NRGs and TME in BRCA by immunohistochemistry, further mechanism experiments are necessary to explore their biological functions.

## Conclusions

In summary, we pioneered the construction of an NRG-based risk model and identified high- and low-risk patients, showing heterogeneity in their functional status, immune microenvironment, genomic variant events, and clinical outcomes. In addition, the constructed risk model could be employed to predict BRCA patient sensitivity towards immunotherapy and chemotherapy. Overall, these results are expected to advance the understanding of necroptosis in tandem with the clinical management and precise treatment for BRCA.

## Data availability statement

The original contributions presented in the study are included in the article/**Supplementary Material**. Further inquiries can be directed to the corresponding author.

## Author contributions

QZ conceived and designed the whole project and drafted the manuscript and was the co- first author. YX and LS analyzed the data and wrote the manuscript. XY carried out data interpretations and helped data discussion. LW provided specialized expertise and collaboration in data analysis. All authors read and approved the final manuscript.

## Funding

This work was granted by the 2018 Clinical Medical Science and Technology Development Fund Project of Jiangsu University (No. JLY20180103).

## Acknowledgments

We thank all the participants who supported our study. In particular, thanks to the TCGA database and GEO database for the analytical data.

## Conflict of interest

The authors declare that the research was conducted in the absence of any commercial or financial relationships that could be construed as a potential conflict of interest.

## Publisher’s note

All claims expressed in this article are solely those of the authors and do not necessarily represent those of their affiliated organizations, or those of the publisher, the editors and the reviewers. Any product that may be evaluated in this article, or claim that may be made by its manufacturer, is not guaranteed or endorsed by the publisher.

## Supplementary material

Supplementary Figure 1 The NRG related risk model in GEO cohort **(A)**. KM survival curves for the high- and low-risk groups in the GEO cohort **(B)**. Survival status of patients and expression of marker genes in GEO cohort; **(C)**.1-,3-,5-,and8-year ROC curves for the RiskScore in GEO cohort.

Supplementary Figure 2 Validation of the treatment decisions in GEO cohort **(A)**. Boxplot showing predicted IC50 values of four commonly used drugs between the two risk groups in the GEO cohort; **(B)**. TIDE algorithm for predicting immunotherapeutic responses of two risk groups in the GEO cohort; **(C)**.Subclass mapping for predicting sensitivity to anti-PD1 and anti-CTLA4 treatment in patients belonging to the high-risk and low-risk groups
