# Supplementary figures and images for: Identification of a novel necroptosis-related classifier to predict prognosis and guide immunotherapy in breast invasive carcinoma
Source: Front Oncol. 2022 Sep 5;12:852365. doi: 10.3389/fonc.2022.852365 (PMC9484550; doi:10.3389/fonc.2022.852365)

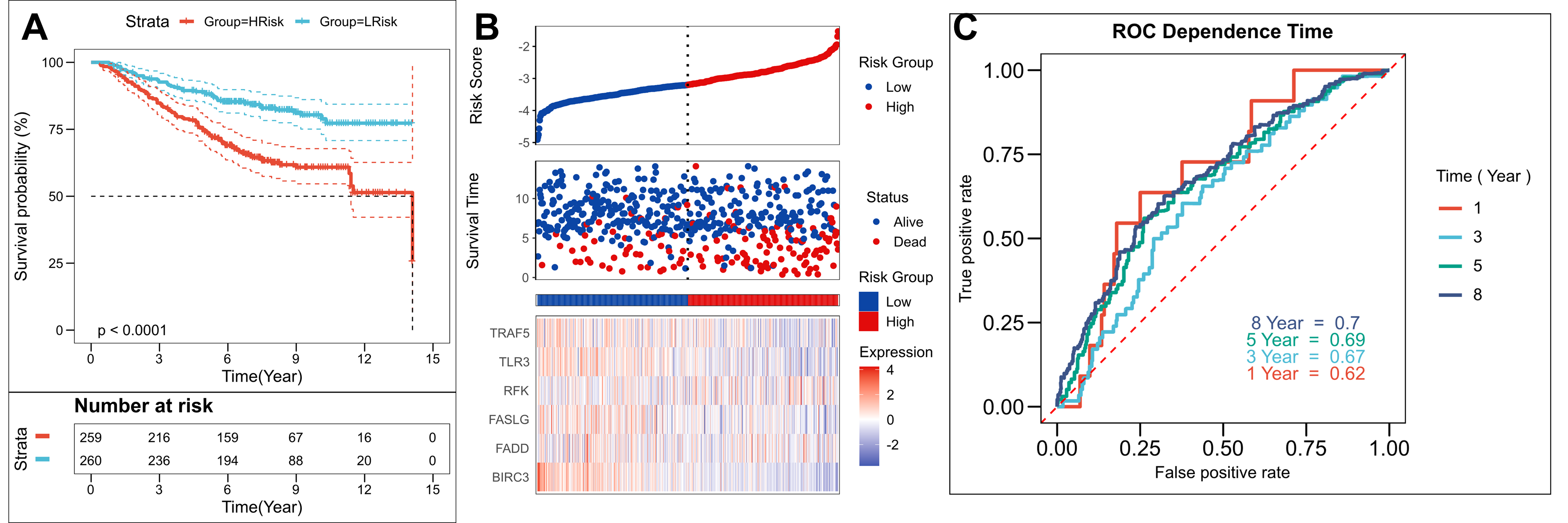

Supplement: Supplementary Figure 1 — The NRG related risk model in GEO cohort (A). KM survival curves for the high- and low-risk groups in the GEO cohort (B). Survival status of patients and expression of marker genes in GEO cohort; (C).1-,3-,5-,and8-year ROC curves for the RiskScore in GEO cohort. [file Image_1.tif]

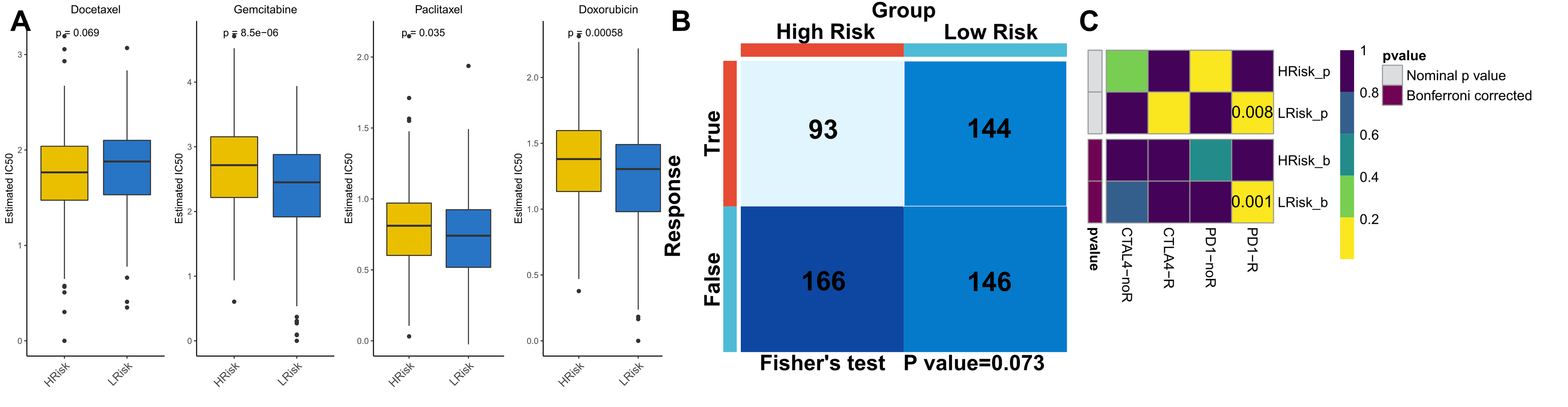

Supplement: Supplementary Figure 2 — Validation of the treatment decisions in GEO cohort (A). Boxplot showing predicted IC50 values of four commonly used drugs between the two risk groups in the GEO cohort; (B). TIDE algorithm for predicting immunotherapeutic responses of two risk groups in the GEO cohort; (C).Subclass mapping for predicting sensitivity to anti-PD1 and anti-CTLA4 treatment in patients belonging to the high-risk and low-risk groups [file Image_2.tif]
